# Hypothermia and Postconditioning after Cardiopulmonary Resuscitation Reduce Cardiac Dysfunction by Modulating Inflammation, Apoptosis and Remodeling

**DOI:** 10.1371/journal.pone.0007588

**Published:** 2009-10-26

**Authors:** Patrick Meybohm, Matthias Gruenewald, Martin Albrecht, Kai D. Zacharowski, Ralph Lucius, Karina Zitta, Alexander Koch, Nguyen Tran, Jens Scholz, Berthold Bein

**Affiliations:** 1 Department of Anaesthesiology and Intensive Care Medicine, University Hospital Schleswig-Holstein, Kiel, Germany; 2 Clinic of Anaesthesiology, Intensive Care Medicine and Pain Therapy, University Hospital Frankfurt, Frankfurt am Main, Germany; 3 Institute of Anatomy, Christian-Albrechts-University, Kiel, Germany; University of Giessen Lung Center, Germany

## Abstract

**Background:**

Mild therapeutic hypothermia following cardiac arrest is neuroprotective, but its effect on myocardial dysfunction that is a critical issue following resuscitation is not clear. This study sought to examine whether hypothermia and the combination of hypothermia and pharmacological postconditioning are cardioprotective in a model of cardiopulmonary resuscitation following acute myocardial ischemia.

**Methodology/Principal Findings:**

Thirty pigs (28–34 kg) were subjected to cardiac arrest following left anterior descending coronary artery ischemia. After 7 minutes of ventricular fibrillation and 2 minutes of basic life support, advanced cardiac life support was started according to the current AHA guidelines. After successful return of spontaneous circulation (n = 21), coronary perfusion was reestablished after 60 minutes of occlusion, and animals were randomized to either normothermia at 38°C, hypothermia at 33°C or hypothermia at 33°C combined with sevoflurane (each group n = 7) for 24 hours. The effects on cardiac damage especially on inflammation, apoptosis, and remodeling were studied using cellular and molecular approaches. Five animals were sham operated. Animals treated with hypothermia had lower troponin T levels (p<0.01), reduced infarct size (34±7 versus 57±12%; p<0.05) and improved left ventricular function compared to normothermia (p<0.05). Hypothermia was associated with a reduction in: (i) immune cell infiltration, (ii) apoptosis, (iii) IL-1β and IL-6 mRNA up-regulation, and (iv) IL-1β protein expression (p<0.05). Moreover, decreased matrix metalloproteinase-9 activity was detected in the ischemic myocardium after treatment with mild hypothermia. Sevoflurane conferred additional protective effects although statistic significance was not reached.

**Conclusions/Significance:**

Hypothermia reduced myocardial damage and dysfunction after cardiopulmonary resuscitation possible via a reduced rate of apoptosis and pro-inflammatory cytokine expression.

## Introduction

Although initial return of spontaneous circulation (ROSC) from cardiac arrest is achieved in about 30–40% of cases, only 10–30% of these patients admitted to the hospital will be discharged with good outcome [1]. Organ dysfunction following successful cardiopulmonary resuscitation (CPR) has mainly been attributed to the so called ‘post-resuscitation disease’ [2]. Post-resuscitation myocardial dysfunction is a critical issue, and has been reported in 45% to 60% of successfully resuscitated patients [3], [4]. Mild therapeutic hypothermia has emerged as an effective strategy to reduce neurological impairment after successful CPR [5]. The effects of mild hypothermia on post-resuscitation myocardial dysfunction, however, are not clear. The mismatch between early survival and final outcome emphasizes the importance of standardized post-resuscitation care including hypothermia. Specifically, pharmacological postconditioning may offer an attractive opportunity to further ameliorate damage to the myocardium in the post-resuscitation period. While pharmacological postconditioning with volatile anesthetics may reduce myocardial injury after acute myocardial infarction [6], its protective properties have not yet been investigated in the context of postresuscitation care. Because the majority of patients experience cardiac arrest due to myocardial ischemia [4], and because this scenario has only been considered in few animal experiments, our study is based on an experimental porcine model of cardiac arrest following acute coronary artery ischemia reflecting a realistic clinical setting. We hypothesized that hypothermia attenuates myocardial injury in this model of global ischemia/reperfusion following coronary artery occlusion. We further hypothesized that the volatile anesthetic sevoflurane - when administered during reperfusion after successful CPR - confers additional organ-protective effects.

## Methods

The project was approved by the Animal Investigation Committee of the University Schleswig-Holstein, Campus Kiel, Germany, and animals were managed in accordance with the guidelines of the University Schleswig-Holstein, Campus Kiel, Germany, and the Utstein-style guidelines [7]. All animals received human care in compliance with the “Guide for the Care and Use of Laboratory Animals” published by the National Institute of Health (NIH Publication No. 88.23, revised 1996). Fully detailed methods and protocols are available in the online supporting information files - Methods S1, Table S1 and Table S2.

### Animals

This is an experimental study on 40 healthy pigs aged 3–4 months of both gender, weighing 28 to 34 kg. Pigs received a total intravenous anesthesia (TIVA; continuous infusion of 4–8 mg/kg/h propofol and 0.3 µg/kg/h sufentanil), and were ventilated for entire study period. Depth of anesthesia was judged according to blood pressure, heart rate and bispectral index (BISXP, Aspect Medical Systems, Natick, MA). Five animals served as sham controls, which were anesthetized with TIVA and treated identically until the end of the experiment except that neither myocardial ischemia was induced nor CPR was performed.

### Experimental protocol

**Figure 1:**
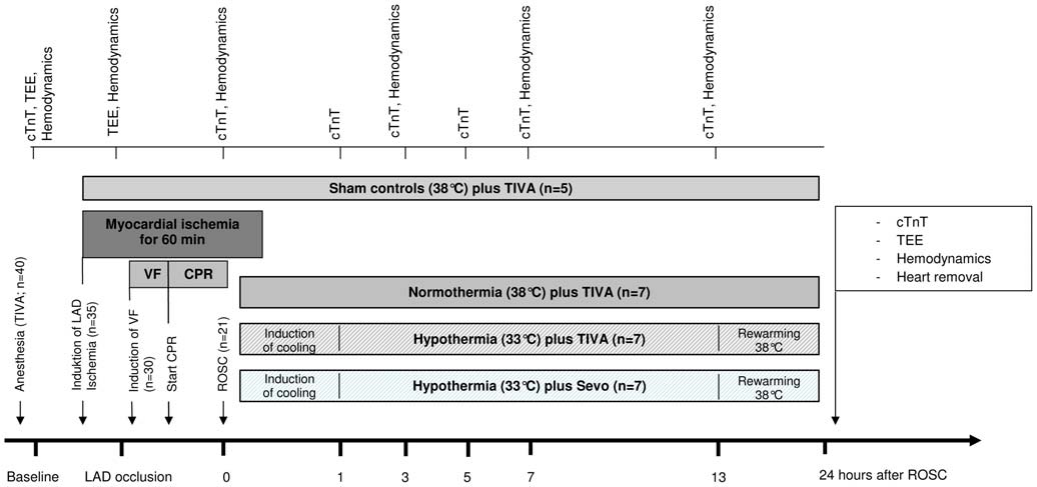
Experimental time line. Thirty pigs were subjected to cardiac arrest following left anterior descending (LAD) coronary artery ischemia. After 7 minutes of ventricular fibrillation (VF), pigs were resuscitated (CPR). After successful return of spontaneous circulation (ROSC; n = 21), coronary perfusion was reestablished after 60 minutes of occlusion, and animals were randomized either to normothermia at 38°C, hypothermia at 33°C or hypothermia at 33°C combined with sevoflurane (each group n = 7) for 24 hours. Five animals were sham operated. cTnT indicates cardiac troponin T; TEE, transesophageal echocardiography.

The experimental time line is presented in figure 1. Thirty-five pigs underwent acute myocardial ischemia according to the technique as previously described [8]. Five pigs fibrillated spontaneously following left anterior descending (LAD) coronary artery occlusion that were excluded from further analysis. Thirty pigs were subjected to cardiac arrest twenty minutes after LAD occlusion. Ventricular fibrillation was electrically-induced by an alternating current of 5 to 10 V in a standardized manner, and mechanical ventilation was discontinued. After a 7-minutes non-intervention interval of untreated ventricular fibrillation, basic life support-CPR was simulated for 2 minutes applying external manual chest compressions at a rate of 100 per minute, and a compression-to-ventilation ratio of 30∶2. Subsequently, advanced cardiac life support was started according to the current AHA guidelines. ROSC was defined as maintenance of an unassisted pulse and a systolic aortic blood pressure of ≥60 mm Hg lasting for ten consecutive minutes according to the Utstein-style guidelines [7]. Coronary perfusion was re-established after 60 minutes of occlusion. After ROSC, animals were randomized either to normothermia (38°C) plus TIVA (NT), hypothermia (33°C) plus TIVA (HT), or hypothermia (33°C) combined with sevoflurane at 1.0 MAC (2.0 Vol % end-tidal) (HT+SEV). Since hypothermia was shown to increase blood concentrations of propofol by about 30% [9], we reduced continuous infusion of propofol during hypothermia targeting bispectral index values below 60. Body core temperature was monitored continuously by the arterial catheter, and normothermic body temperature was maintained at 38.0°C with a heating blanket. Hypothermia was induced by a cooling device (Icy catheter and CoolGard 3000; Alsius Corp, Irvine, USA) that was introduced into the femoral vein. According to the landmark study by Bernard et al. [10] we used a target body temperature of 33°C for 12 hours. Thereafter, rewarming was initiated (0.5°C per hour). During the post-resuscitation period, animals received crystalloid infusions to keep central venous pressure above 8 mm Hg and mean arterial blood pressure above 50 mm Hg. If this first step failed, additional norepinephrine was administered to keep mean arterial blood pressure above 50 mm Hg. We further aimed at serum glucose levels less than 150 mg/dL by intermittent insulin bolus administration. Animals were killed by an overdose of sufentanil, propofol and potassium chloride 24 hours after ROSC, and tissue samples of the ischemic and non-ischemic myocardium were collected and then both snap-frozen in liquid nitrogen (stored at −80°C) and fixed in 4% formaldehyde solution followed by embedding into paraffin blocks, respectively.

### Echocardiography and hemodynamics

Two-dimensional and pulsed-waved Doppler transesophageal echocardiography (TEE) was performed by a single experienced examiner using a Vivid i- Cardiovascular Ultrasound System (GE Healtcare, Munich, Germany) with an omniplane TEE probe as described before [11]. Hemodynamic data were determined at baseline and repetitively up to 24 hours after ROSC.

### Assessment of infarct size

Myocardial infarct size was determined 24 hours after ROSC as previously described [8]. In addition, blood samples were collected at baseline and repetitively up to 24 hours after ROSC to determine cardiac troponin T (cTnT).

### Histology and apoptosis

Paraffin-embedded slides of ischemic myocardium were stained with hematoxylin and eosin. Caspases are proteases involved in the apoptotic and inflammatory cascade, and in particular, caspase 3 is a central mediator of the apoptotic cascade [12]. To quantify apoptosis, we determined uncleaved procaspase 3 by western blotting in a first step, and performed immune fluorescence in a second step to depict active caspase 3 in the ischemic myocardial tissue.

### Myeloperoxidase assay

Myeloperoxidase, an enzyme present in leukocytes, is an index of tissue immune cell infiltration and was determined in ischemic and non-ischemic myocardial tissue samples as previously described [13].

### Quantitative real-time RT-PCR

Transcript levels of the cytokines interleukin (IL)-1β, IL-6, IL-10, tumor necrosis factor-α and intercellular adhesion molecule-1 were investigated in both ischemic and non-ischemic myocardium and compared with sham control animals.

### Enzyme-linked immunosorbent assay (ELISA)

Protein concentrations of IL-1β were determined by a swine specific ELISA (BioSource International, Inc. Camarillo, USA) in homogenates of frozen tissues according to the manufacturer's protocol. All experiments were carried out in duplicates.

### Gelatine zymography- activity of MMP-9 and MMP-2

Matrix metalloproteinases (MMPs) are typical enzymes involved in myocardial remodeling. To investigate the activity of MMP-9 and MMP-2 in ischemic and non-ischemic myocardial tissue, gelatine zymography employing myocardial tissue was performed as described previously [14].

### *In-vitro* cell culture experiments

To evaluate the regulatory effects of IL-1β on MMPs activity and cell proliferation - two major events in tissue remodeling – we used the well characterized human fibroblast cell line HT-1080 [15] for further *in-vitro* cell culture experiments.

### Statistical analysis

Sample size was calculated based on a previous investigation [8] to detect a reduction in myocardial infarct size of about 50%. For an α of 0.05 and a power of 80%, we calculated a sample size of 5 animals in each group. To account for animals who did not achieve ROSC or died during the postresuscitation period, we used 10 animals per group for LAD occlusion and CPR. Statistics were performed using commercially available statistics software (GraphPad Prism version 5.02 for Windows, GraphPad Software, San Diego, CA). Survival rates were compared using Fisher's exact test. Date were analyzed by one-way repeated measures analysis of variance (ANOVA), and in cases where significant differences were observed, adjusted for multiple comparisons (Bonferroni). Variables are expressed as mean ± SD or scatter plots unless otherwise specified. Statistical significance was considered at a two-sided p value of ≤0.05.

## Results

### Survival of animals

Twenty-one animals were successfully resuscitated. Detailed resuscitation data are presented in table 1. In the NT group, 5 out of 7 animals survived for 24 hours compared to all animals in the HT and HT+SEV group (p = 0.46 vs. NT). Two animals of the NT-group died due to hemodynamic instability during the post-resuscitation period.

**Table 1:** Cardiopulmonary resuscitation data.

	NT	HT	HT+SEV	*p* values
ROSC rate [n]	7/10	7/10	7/10	-
CPR time to successful resuscitation [min]	9.7±2.8	10.3±3.4	10.5±3.1	0.939
Cumulative epinephrine dose [μg/kg]	100±44	101±47	93±33	0.828
Cumulative vasopressin dose [IU/kg]	0.8±0.2	0.8±0.3	0.8±0.3	0.897
Cumulative defibrillation energy [J]	755±420	703±413	795±199	0.854
CorPP 10 [mm Hg]	31±9	26±11	28±6	0.559
CorPP 15 [mm Hg]	39±28	40±25	38±24	0.890
Time to target temperature 33°C [min]	-	47±10	45±15	-

Return of spontaneous circulation (ROSC), cardiopulmonary resuscitation (CPR) time to successful resuscitation, cumulative epinephrine and vasopressin dose, cumulative defibrillation energy, coronary perfusion pressure (CorPP) 10 and 15 minutes after induction of ventricular fibrillation, and induction time to target temperature of 33°C. NT indicates normothermia; HT, hypothermia; HT+SEV, hypothermia combined with sevoflurane. Data are mean±SD.

### Myocardial damage

**Figure 2:**
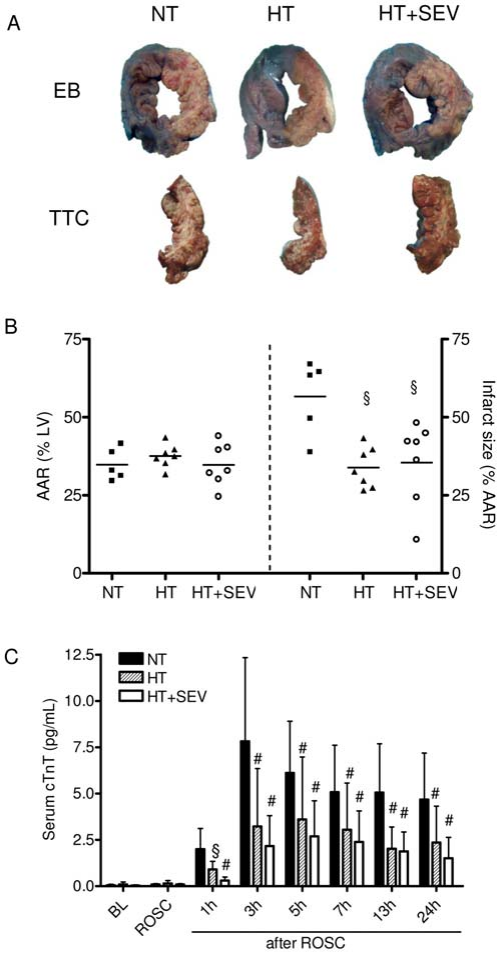
Myocardial damage. A) Evans blue (EB) staining to determine the area at risk (AAR; three images in the top row, corresponding to pigs subjected to normothermia (NT), hypothermia (HT), or hypothermia and sevoflurane (HT+SEV), respectively). AAR was subjected to triphenyltetrazolium chloride (TTC) staining to determine infarct size (corresponding images in the lower row). B) AAR expressed as percentage of left ventricle (LV). Infarct size expressed as percentage of the AAR. Data are expressed as dot plots and mean. C) Serum cardiac troponin T (cTnT) release at baseline (BL), after return of spontaneous circulation (ROSC) and 1, 3, 5, 7, 13 and 24 hours after ROSC. Data are expressed as mean ± SD. §p<0.05, #p<0.01 vs. NT.

Figure 2A depicts corresponding images of staining to determine ischemic (AAR), non-ischemic (AANR), and infarcted myocardium after 24 hours. The AAR did not differ between groups. Infarct size was 57±12% in the NT group. HT and HT+SEV significantly reduced infarct size to 34±7% and 35±13%, respectively (p<0.05; Figure 2B). Animals treated with HT had significantly lower peak serum levels of cTnT (3.6±3.3 pg/mL) compared with the NT group (7.8±4.5 pg/mL; p<0.01). HT+SEV further attenuated peak serum cTnT (2.7±1.9 pg/mL) although significance was not reached compared to HT (Figure 2C).

### Echocardiography and hemodynamics

**Figure 3:**
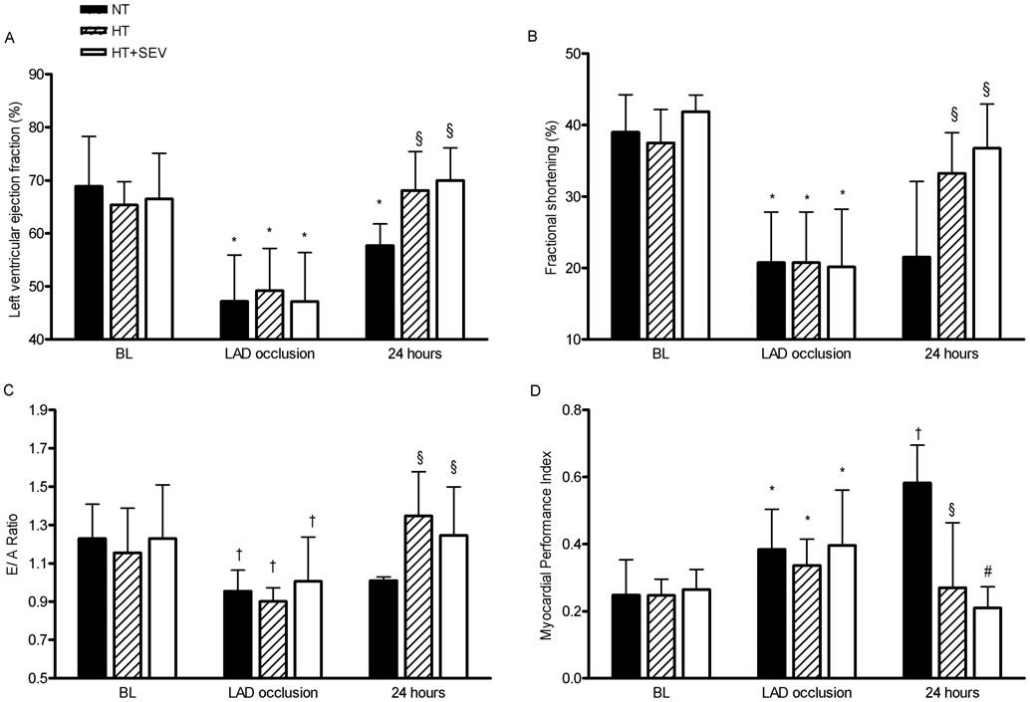
Transesophageal echocardiography. A) Left ventricular ejection fraction, B) fractional shortening, C) E/A ratio and D) myocardial performance index at baseline (BL), after left anterior descending (LAD) coronary artery occlusion, and 24 hours after return of spontaneous circulation in the normothermia (NT), hypothermia (HT) and hypothermia and sevoflurane (HT+SEV) group. *p<0.05, †p<0.01, ‡p<0.001 vs. baseline; §p<0.05, #p<0.01 vs. NT.

Analysis of TEE data revealed impaired left ventricular ejection fraction (p<0.05), fractional shortening (p<0.05), E/A ratio (p<0.01) and myocardial performance index (p<0.05) before cardiac arrest as a result of acute LAD occlusion. While myocardial function remained impaired compared to BL in the NT group (p<0.05), echocardiographic variables reached baseline values in the HT and HT+SEV group 24 hours after ROSC, respectively, and were significantly improved compared to NT (p<0.05 vs. NT, Figure 3). Further systemic hemodynamic variables are presented in table 2. Heart rate, mean arterial blood pressure and cardiac index did not significantly differ between groups corrected for repeated time measurements defining statistical significance at a p value of ≤0.007. Cumulative crystalloid fluid load and cumulative norepinephrine dose were not significantly different between groups 24 hours after ROSC [NT (volume load (p = 0.540); norepinephrine dose (p = 0.812): 4241±1244 mL; 4.4±1.6 mg), HT (3987±932 mL; 4.9±2.1 mg), HT+SEV (4627±1056 mL; 5.1±1.8 mg)].

**Table 2:** Hemodynamic data.

	NT	HT	HT+SEV	*p* values
**Baseline**				
HR, beats/minute	107±21	105±14	96±15	0.342
MAP, mm Hg	65±13	70±11	71±13	0.314
ETco_2_, mm Hg	40±5	36±4	37±5	0.180
CI, L/min/m^2^	7.4±1.8	6.8±1.3	7.4±1.7	0.580
**Ischemia**				
HR, beats/minute	103±14	99±17	95±18	0.609
MAP, mm Hg	57±6	57±7	63±13	0.469
ETco_2_, mm Hg	40±±3	40±3	36±5	0.050
CI, L/min/m^2^	5.1±0.7	5.1±0.8	5.4±1.1	0.662
**ROSC**				
HR, beats/minute	94±18	99±33	96±21	0.988
MAP, mm Hg	55±6	65±21	60±8	0.225
ETco_2_, mm Hg	39±9	42±4	39±7	0.363
CI, L/min/m^2^	4.4±0.5	4.5±1.9	4.6±1.0	0.982
**3 hours**				
HR, beats/minute	131±19	147±8	117±19	0.005
MAP, mm Hg	58±6	58±6	55±6	0.799
ETco_2_, mm Hg	39±5	36±4	35±4	0.080
CI, L/min/m^2^	6.3±1.2	5.9±0.7	6.4±1.7	0.819
**7 hours**				
HR, beats/minute	131±17	140±22	127±20	0.821
MAP, mm Hg	58±4	59±12	61±8	0.820
ETco_2_, mm Hg	37±7	35±4	35±2	0.731
CI, L/min/m^2^	6.2±0.4	5.5±1.1	7.6±1.2	0.010
**13 hours**				
HR, beats/minute	135±20	121±44	116±17	0.627
MAP, mm Hg	53±4	58±6	60±10	0.474
ETco_2_, mm Hg	38±7	36±3	36±7	0.636
CI, L/min/m^2^	5.7±0.4	5.7±0.4	7.0±1.4	0.175
**24 hours**				
HR, beats/minute	154±25	143±19	124±24	0.297
MAP, mm Hg	46±6	57±12	54±4	0.249
ETco_2_, mm Hg	37±4	41±1	37±2	0.180
CI, L/min/m^2^	5.6±0.2	7.1±1.6	8.7±1.8	0.139

Hemodynamic data were determined at baseline, after 20 minutes of coronary occlusion (Ischemia), following return of spontaneous circulation (ROSC) and 3, 7, 13, and 24 hours after ROSC (Mean±SD). HR indicates heart rate; MAP, mean arterial blood pressure; ETco_2_, end-tidal carbon dioxide, CI, cardiac index is calculated as the ratio of cardiac output/body surface area (body surface area  =  0.0734*(body weight in kg)^0.656^) [36]. Considering the repeated measures factor, statistical significance was considered at a two-sided p value of ≤ 0.007.

### Infiltration of immune cells and apoptosis

**Figure 4:**
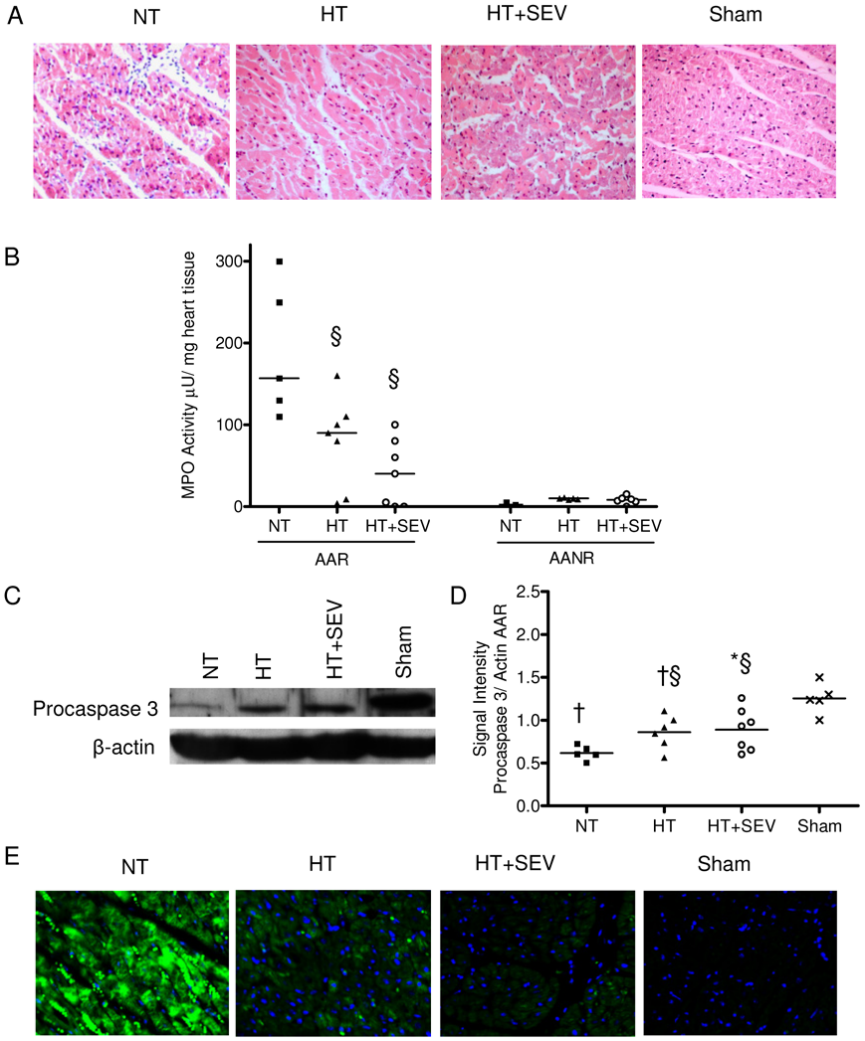
Myocardial infiltration of immune cells and apoptosis. A) Representative hematoxylin and eosin staining of the ischemic myocardium (400×magnification) corresponding to pigs subjected to normothermia (NT), hypothermia (HT), hypothermia and sevoflurane (HT+SEV) or sham control are shown. Myocardial samples from all animals used in the study were processed for histopathological analysis. B) Quantification of myeloperoxidase (MPO) activity in myocardial tissue for area at risk (AAR) and area at no risk (AANR). C, D) Myocardial protein expression of procaspase-3 and β-actin were determined by western blotting from all animals used in the study. Representative blots of pigs subjected to NT, HT, HT+SEV, and sham control are shown. E) Representative anti-caspase 3 immunfluorescence staining of the AAR (400×magnification). Data are expressed as dot plots and mean. *p<0.05, †p<0.01 vs. Sham. §p<0.05 vs. NT.

HT and HT+SEV animals exhibited reduced infiltration of immune cells in the ischemic myocardium (Figure 4A). MPO activity in ischemic tissue (AAR) was significantly decreased in the HT (80±50 µU/mg) and HT+SEV group (40±40 µU/mg) compared to the NT group (190±80 µU/mg; p<0.05), confirming the morphological results (Figure 4B). Procaspase 3 was most markedly decreased in the NT group (0.62±0.08 a.u.) compared to sham control (1.25±0.18 a.u.) indicating cleavage of inactive procaspase 3 to caspase 3 (p<0.001; Figure 4C, D). To confirm results of western blotting we used immune fluorescence in a second step to depict active caspase 3 that was clearly attenuated in the HT and HT+SEV group compared to NT (Figure 4E).

### Myocardial inflammation

**Figure 5:**
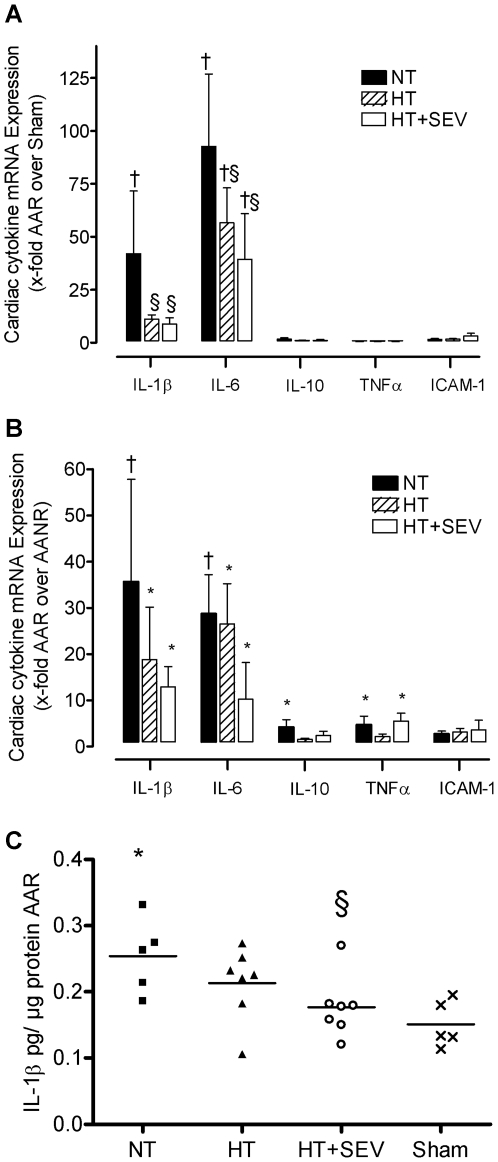
Myocardial cytokine expression. Transcript levels of the cardiac cytokines interleukin (IL)-1β, IL-6, IL-10, tumor necrosis factor (TNF)-α and intercellular adhesion molecule (ICAM)-1 were determined by quantitative RT-PCR (A, B), and protein concentrations of IL-1β were determined by a swine specific enzyme-linked-immunosorbent assay (C). AAR indicates area at risk; AANR, area at no risk; NT, normothermia; HT, hypothermia; HT+SEV, hypothermia combined with sevoflurane. Data are expressed as mean±SD (A, B) and dot plots/mean (C), respectively. *p<0.05, †p<0.01 vs. Sham. §p<0.05 vs. NT.

NT resulted in a significant 42±29-fold increase of IL-1β and 93±34-fold increase of IL-6 mRNA-levels in ischemic myocardial tissue (AAR) compared with sham control (p<0.01). HT and HT+SEV significantly reduced up-regulation of IL-1β (HT 11±2-fold; HT+SEV 9±3-fold; p<0.05) and IL-6 mRNA-levels (HT 57±16-fold; HT+SEV 39±21-fold; p<0.05; Figure 5A). Comparing ischemic (AAR) with non-ischemic myocardial tissue (AANR), NT resulted in a significant up-regulation of IL-1β (36±22-fold) and IL-6 (29±8-fold) mRNA-levels (p<0.01) that was significantly attenuated by HT and HT+SEV for IL-1β (HT 19±11-fold; HT+SEV 13±4-fold) and IL-6 mRNA-levels (HT 26±9-fold; HT+SEV 10±8-fold; p<0.05; Figure 5B). IL-1β protein concentration was decreased in the HT (0.21±0.06 pg/µg) and HT+SEV group (0.18±0.05 pg/µg) compared to the NT group (0.25±0.06 pg/µg protein; p<0.05 NT versus HT+SEV; Figure 5C).

### Myocardial MMP-9 and MMP-2 activity

**Figure 6:**
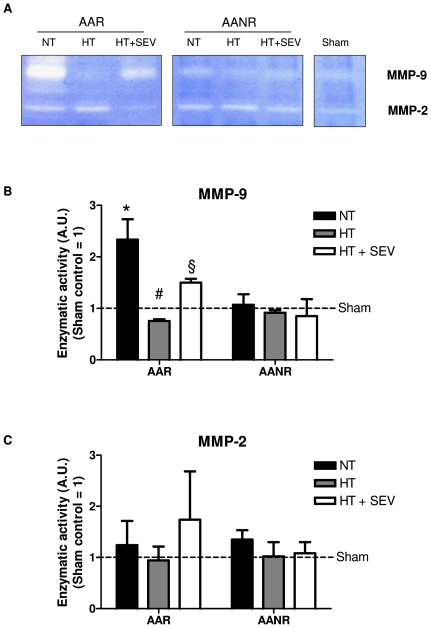
Effects of hypothermia and pharmacological postconditioning on MMP-9 and MMP-2 activity. Activity of matrix metalloproteinase (MMP)-9 and MMP-2 was evaluated by gelatine zymography employing ischemic (AAR) and non-ischemic (AANR) myocardial tissue of the same animal. A) One representative gel out of three is shown. White bands represent areas of enzymatic activity of MMP-9 and MMP-2, respectively. Mean intensity of the zymography bands was normalized to sham control (dashed line) separately for MMP-9 (B) and MMP-2 (C). AAR indicates area at risk; AANR, area at no risk; NT, normothermia; HT, hypothermia; HT+SEV, hypothermia combined with sevoflurane. Data are expressed as mean±SD. All experiments were repeated three times. *p<0.001 vs. Sham, §p<0.05, #p<0.001 vs. NT.

HT and HT+SEV significantly reduced MMP-9 activity compared to NT (HT p<0.001; HT+SEV p<0.05), however, HT+SEV was not superior to HT alone (Figure 6A and B). Although HT and HT+SEV tended to regulate individual MMP-2 activity, this tendency could not be confirmed by densitometry employing higher numbers of animals per group (Figure 6C).

## Discussion

Circulatory failure and myocardial dysfunction resulting from cardiac arrest largely contribute to morbidity and mortality after initially successful CPR [16]. We showed that (i) mild hypothermia reduced myocardial damage and dysfunction after CPR and myocardial ischemia most probably due to attenuation of immune cell infiltration, apoptosis and pro-inflammatory cytokine expression, and (ii) pharmacological postconditioning with sevoflurane may confer additional protective effects on myocardium.

Coronary heart disease including acute myocardial ischemia is the major cause of out-of-hospital cardiac arrest [4], [17]. To the best of our knowledge, however, there have only been a few animal studies considering this complex scenario of myocardial ischemia as the predecessor to cardiac arrest [18], [19]. Therefore, we established a porcine model of cardiac arrest following myocardial ischemia with prearrest impaired left ventricular function to mimic a realistic scenario of out-of-hospital cardiac arrest. Prearrest impaired cardiac function due to acute myocardial ischemia has been found to result in a substantially worse survival compared with prearrest normal cardiac function [3].

### Effects of hypothermia on myocardial damage and function

From our data, we conclude that HT initiated after ROSC reduced myocardial damage compared to NT. Analysis of echocardiographic variables revealed a deterioration of left ventricular function due to LAD occlusion before cardiac arrest, that remained declined in the NT group in the post-resuscitation period, but improved in the HT group 24 hours after ROSC, respectively. The difference in survival found - although not statistically significant possibly due to an inadequate sample size - may be rooted in this improved myocardial function. Following two underlying mechanisms may be considered: i) amelioration of post-resuscitation myocardial stunning by hypothermia that has recently been demonstrated in a porcine model of non-infarct cardiac arrest [20], and ii) myocardial infarct sparing effects by hypothermia that has also been noted [21].

In terms of available clinical evidence, mild hypothermia may be a promising new therapeutic strategy to prevent myocardial reperfusion injury after cardiac arrest in the post-resuscitation period. Its use in daily clinical routine, however, is controversial at present [22]. Very recently, a retrospective subgroup analysis of the ‘Hypothermia after Cardiac Arrest’ trial revealed a favorable trend for early cooling in patients with cardiac arrest following myocardial ischemia [23]. Further, Wolfrum et al. demonstrated that the combination of rapid induction of mild hypothermia and percutaneous coronary intervention is feasible, safe, and beneficial after cardiac arrest, in particular in patients with acute myocardial ischemia [24]. In contrast, clinical studies of therapeutic hypothermia in patients with myocardial infarction but without cardiac arrest have not shown any beneficial effects [25], [26]. Obviously, the effects of mild hypothermia may differ between patients who had undergone resuscitation and patients with myocardial ischemia but without cardiac arrest.

In our experimental study, HT reduced immune cell accumulation and decreased myocardial apoptosis by reduced cleavage of procaspase 3 to active caspase 3 in the ischemic myocardium compared to NT. In addition, we found HT to be associated with attenuated up-regulation of IL-1β and IL-6 mRNA expression, and reduced IL-1β protein expression in ischemic myocardial tissue. Interestingly, IL-6 production is associated with negative inotropic effects and myocardial dysfunction that may be related to enhanced production of nitric oxide [27]. The key factors of myocardial ischemia/reperfusion injury are accumulation of neutrophils and the release of various inflammatory cytokines that are generated locally in the heart and contribute to up-regulation of cell-adhesion molecules, cardiac functional depression and apoptosis [28].

After substantial myocardial ischemia, major tissue remodeling occurs aiming to restore the structural architecture and cardiac function. These remodeling events go in parallel with an increased expression and activity of MMPs in the post-infarcted myocardium [29]. Regarding the effect of hypothermia on myocardial tissue remodeling, we found that hypothermia reduced MMP-9 activity in ischemic myocardial tissue, whereas the effects on MMP-2 activity were indistinguishable. Since IL-1β and other pro-inflammatory cytokines may also be actively involved in the regulation of matrix remodeling processes after myocardial ischemia [30], the issue of tissue remodeling and the regulatory effect of IL-1β on MMPs activity could be of outstanding importance. Interestingly, preliminary *in-vitro* data from our group points towards positive regulatory effects of IL-1β on MMP-2 and MMP-9 expression and activity (Supporting information files - Results S1 and Figure S1). Two studies further demonstrated a central role of IL-1β as a ‘pro-fibrotic’ factor after myocardial ischemia: (i) IL-1 receptor type 1 knockout mice showed attenuated myocardial MMP-2 and MMP-3 expression and decreased fibrotic remodeling [30], and (ii) administration of a IL-1 receptor antagonist ameliorated remodeling processes in the infarcted myocardium [31]. These data suggest that the hypothermia-induced attenuation of IL-1β expression in the present study may possibly affect cardiac MMP-2 and MMP-9 activity facilitating tissue remodeling following CPR and myocardial ischemia.

### Additional effects of pharmacological postconditioning

Most experimental studies have documented improved functional performance when organprotective agents were given before the insult [32]. In patients with cardiac arrest, however, pretreatment is virtually impossible because of the unpredictable onset. Therefore, as in our study, protective interventions should be started after the initiation of global reperfusion, when significant damage has already occurred. In this context, pharmacological postconditioning with volatile anesthetics in addition to mild hypothermia - currently recommended by the International Liaison Committee on Resuscitation for patients with out of hospital cardiac arrest - may offer an attractive opportunity to further ameliorate organ damage in the post-resuscitation period. In our study, sevoflurane administered instead of propofol during reperfusion after successful CPR tended to further attenuate serum cTnT release, MPO activity, apoptosis and local myocardial inflammatory response. These results, however, did not reach statistical significance possibly due to an inadequate sample size rather than a true difference was absent. Zhao and coworkers demonstrated that postconditioning with sevoflurane resulted in massive salvage of the myocardium and reduction of infarct size by 45% [33]. Others have reported that protective effects of sevoflurane are mediated by mitochondrial K_ATP_ channel opening, reduction of Ca^2+^-loading, and anti-inflammatory mechanisms [34].

With respect to our experimental setting, hypothermia alone may possess such potent organ protective properties compared to normothermia, that an additional effect of sevoflurane could not be revealed in the present study. Moreover, potential protective effects of volatile anesthetics depend on energy-dependent signal transduction, e.g. protein synthesis and phosphorylation [35], that may be affected by hypothermia-induced decrease of global metabolic rate as well as suppression of protein synthesis.

### Limitations

Although we used a porcine model of cardiac arrest following myocardial ischemia reflecting this common clinical scenario, there are several points that need to be addressed in future studies: (i) both long-term survival and neurological outcome were not evaluated because of limitations posed by governmental regulations. (ii) Induction of hypothermia has been started after reperfusion of global ischemia but during myocardial ischemia that may affect cardioprotective properties of hypothermia. However, animal data [21] and subgroup analysis of human trials [22] consistently show that hypothermia has to be induced before myocardial reperfusion, and that the protective effect of hypothermia is completely lost if the onset of cooling is delayed into the myocardial reperfusion period. This may be the major difference to global ischemia due to cardiac arrest, where hypothermia proved to be beneficial even when induced after the initiation of reperfusion. (iii) Blinding the investigator was not possible throughout the experiment due to cooling technique, but infarct size, TEE variables, and serum and tissue samples were analyzed in a blinded fashion.

### Conclusions

In conclusion, (i) hypothermia after cardiac arrest following myocardial ischemia reduced myocardial damage, dysfunction, apoptosis, up-regulation of inflammatory cytokines and MMP activity in ischemic myocardium compared to NT. (ii) The volatile anesthetic sevoflurane - when administered during reperfusion after successful CPR - conferred additional protective effects in the above setting without reaching statistical significance. We suggest that sevoflurane in addition to hypothermia may be considered as a protective intervention in the post-resuscitation period.

## Supporting Information

Extended Methods S1 Extended Methods section
(0.11 MB DOC)

Table S1 Primer sequences and TaqMan probes (in-vivo experiment).
(0.06 MB DOC)

Table S2 Primer sequences and amplicon sizes (in-vitro cell culture).
(0.04 MB DOC)

Results S1 Preliminary results as Online Supplement and Figure legends for Supporting Information file - Figure S1.
(0.05 MB DOC)

Figure S1 Effects of IL-1β on cell proliferation and MMPs activity in vitro.
(2.37 MB TIF)
