# Supplementary material for: Hypothermia and Postconditioning after Cardiopulmonary Resuscitation Reduce Cardiac Dysfunction by Modulating Inflammation, Apoptosis and Remodeling
Source: PLoS One. 2009 Oct 26;4(10):e7588. doi: 10.1371/journal.pone.0007588 (PMC2764338; doi:10.1371/journal.pone.0007588)
Supplement: Extended Methods S1 — Extended Methods section (0.11 MB DOC) [file pone.0007588.s001.doc]

# **Supporting information file –**

**Methods S1**

**Hypothermia and postconditioning after cardiopulmonary resuscitation reduce cardiac dysfunction by modulating inflammation, apoptosis and remodeling**

**Animal preparation**

All animals (German domestic pigs) received human care in compliance with the ”Guide for the Care and Use of Laboratory Animals” published by the National Institute of Health (NIH Publication No. 88.23, revised 1996). The animals were fasted overnight, but had free access to water. Anesthesia was initiated by intramuscular injection of azaperone (8 mg/kg) and atropine (0.05 mg/kg), and completed by ear vein injection of propofol (1-2 mg/kg) and sufentanil (0.3 µg/kg). After endotracheal intubation, pigs were ventilated with a volume-controlled ventilator (Siemens SV 900C, Erlangen, Germany) and the following setting: a FiO_2_ of 0.3 at 20 breaths/minute, a tidal volume of 8 mL/kg to maintain normocapnia, and a positive end-expiratory pressure of 5 mm Hg. Ventilation was monitored using an inspired/expired gas analyzer that measured oxygen and end-tidal carbon dioxide (suction rate, 200 mL/min; M-PRESTN; Datex-Ohmeda Inc., Helsinki, Finland). Total intravenous anesthesia (TIVA) was maintained by continuous infusion of propofol (4-8 mg/kg/h) and sufentanil (0.3 µg/kg/h); muscle relaxation was achieved by continuous infusion of pancuronium (0.2 mg/kg/h). Ringer’s solution (10 mL/kg/h) was administered continuously throughout the preparation phase to replace fluid loss during instrumentation. Standard leads II and V_5_ electrocardiogram were used to monitor cardiac rhythm and ST-segment changes. Depth of anesthesia was judged according to blood pressure, heart rate and Bispectral Index (BISXP, Aspect Medical Systems, Natick, MA). Pigs do not respond to painful or auditory stimuli under this anesthetic regimen when the paralyzing agent is withheld, and the loading dose of propofol subsides.

A 7F saline filled central venous catheter was inserted in the right internal jugular vein for drug administration. A 4F thermistor-tipped catheter for arterial thermodilution (Pulsion Medical Systems, Munich, Germany) was inserted percutaneously into the right femoral artery. The arterial catheter was connected to the PiCCO system (PiCCO plus, Software Version 6.0, Pulsion Systems, Munich, Germany), and the resulting signal processed to determine mean arterial blood pressure, heart rate, and blood temperature. In addition, the arterial catheter allowed discontinuous measurement of transpulmonary cardiac output by injecting 10 mL ice cold saline into the proximal port of the central venous catheter. The mean of three consecutive measurements randomly assigned to the respiratory cycle was used for determination of cardiac output. Intravascular catheters were attached to pressure transducers (Smiths Medical, Kirchseeon, Germany) that were aligned at the level of the right atrium. All catheters were flushed with isotonic saline containing 5 IU/mL heparin at a rate of 3 mL/h to prevent obstruction.

**Experimental protocol**

Following hemodynamic measurements at baseline, 35 pigs were subsequently subjected to acute myocardial ischemia according to the technique as previously described [1]. Briefly, the left anterior descending coronary artery (LAD) was dissected directly behind the first diagonal branch following a lateral mini-thoracotomy, and was clasped by a vessel-loop. The artery was then occluded by sliding a tubing over the vessel loop and clamping it with a small hemostat. Then, mini-thoracotomy was surgically completely repaired to gain closed chest conditions. To prevent clot formation, the animals received heparin (100 IU/kg) prior to LAD occlusion and induction of cardiac arrest. After a 7-minutes nonintervention interval of untreated ventricular fibrillation, basic life support-CPR was simulated for 2 min. External standard manual closed chest compressions were performed at a rate of 100 per minute with a 50% duty cycle, and a compression depth of 25% of the anterior-posterior diameter of the chest wall. The compression-to-ventilation ratio was 30:2, thus 30 compressions followed by two rescue breaths with a self-inflating bag. Subsequently, advanced cardiac life support was started with one 100 J biphasic defibrillation attempt (M-Series Defibrillators, Zoll Medical Corporation, Chelmsford, USA) according to the one-shock protocol of the current CPR guidelines [2]. Further, ventilations were performed with a semiautomatic ventilator with 100 % oxygen at 20 breaths/minute. All pigs received 45 μg/kg epinephrine and 0.4 U/kg vasopressin alternating as suggested by the AHA guidelines [2]. ROSC was defined as maintenance of an unassisted pulse with a systolic aortic blood pressure of ≥ 60 mm Hg lasting for ten consecutive minutes according to the Utstein-style guidelines [3]. Coronary perfusion was re-established by release of the vessel loop after 60 minutes of occlusion. Since neurological recovery is very unlikely after 30 minutes of normothermic cardiac arrest, CPR was terminated, when resuscitation remained unsuccessful after 23 minutes of CPR. While animals of NT and HT group were anaesthetized with TIVA after ROSC at the same dosage used during the preparation phase, 1.0 MAC sevoflurane (2.0 Vol % end-tidal) and sufentanil (0.3 µg/kg/h) were administered during reperfusion and hypothermia in HT+SEV group. One hour after ROSC, FiO_2_ was reduced to 0.4.

Autopsy was routinely performed for documentation of potential injuries to the thoracic and abdominal cavity during CPR.

**Echocardiography**

Mitral valve inflow velocity pattern was recorded from the midesophageal four-chamber view with the pulsed-wave Doppler sample volume positioned at the tips of the mitral leaflets during diastole. Peak velocities of transmitral inflow were measured in early (E) and late (A) diastole, and E/A ratio was calculated. Left ventricle (LV) outflow tract and the ascending aorta were imaged, and a pulsed wave Doppler was positioned at the LV outflow tract with the sample volume just below the aortic valve for determination of ejection time. Beam position and gain settings were optimized to achieve the greatest amplitude and clarity of the Doppler spectrum. LV ejection time was measured from the beginning to the end of the aortic flow wave. The LV end-systolic and end-diastolic volumes were estimated using long-axis and four-chamber biplane views by tracing the endocardial border including the papillary muscles and the method of discs according to modified Simpson’s rule algorithm. Then, LV ejection fraction (LVEF) was obtained. The transgastric midpapillary short-axis view of the left ventricle (LV) was obtained with the TEE multiplane transducer positioned at 0° to determine end-diastolic and end-systolic diameter to calculate fractional diameter shortening (FS), though standard short-axis view was difficult to achieve in some pigs. The myocardial performance index (MPI) has been developed to reflect systolic and diastolic function [4]. The interval (a) was measured from mitral valve closing to opening, which is equal to the sum of isovolumetric contraction time, ejection time, and isovolumetric relaxation time. LV ejection time (b) was measured from the onset to the end of blood flow in the LV outflow tract. The sum of isovolumetric contraction time and isovolumetric relaxation time was obtained by subtracting (b) from (a). MPI was then calculated as (a- b/ b). All echocardiographic measurements were recorded as the mean of three consecutive cardiac cycles, and were made according to the recommendations of the American Society of Echocardiography [5]. Another experienced echocardiographer blinded to group assignment performed *post hoc* off-line quantitative data analyses.

**Hemodynamic measurements**

Hemodynamic data, including mean arterial blood pressure, heart rate, end-tidal carbon dioxide, and cardiac output were determined at baseline (BL), after 20 minutes of LAD occlusion, following ROSC and 3, 7, 13, and 24 hours after ROSC.

**Assessment of infarct size**

After 24 hours, the LAD was re-occluded, and Evans blue dye (40 mL of 2 % w/v in phosphate-buffered saline) was administered intravenously to distinguish between ischemic (area at risk; AAR) and non-ischemic myocardium (area at no risk; AANR). The left ventricle was cut in six 4-mm slices, digitally photographed, incubated with triphenyl tetrazolium chloride (1% w/v in phosphate-buffered saline at 37°C) and photographed for a second time to determine infarcted tissue, as previously described [1]. Infarct size was expressed as percentage of the AAR in a blinded fashion by an independent investigator.

**Determination of serum markers**

Arterial blood samples were collected at BL, following ROSC and 1, 3, 5, 7, 13, and 24 hours after ROSC. Serum was obtained (centrifuged at 3000*g* for 5 min) and stored at -20°C until determination of cardiac troponin T (cTnT) by an independent laboratory (Institute of Clinical Chemistry, University Hospital Schleswig-Holstein, Campus Kiel, Germany).

**Histology and Apoptosis**

Paraffin-embedded 7 µm slides of ischemic myocardium were stained with hematoxylin and eosin. One investigator blinded to the group assignment evaluated histopathological changes and immune cell infiltration.

Caspases are proteases involved in the apoptotic and inflammatory cascade, and in particular, caspase 3 is a central mediator of the apoptotic cascade [6]. To quantify apoptosis, we determined uncleaved procaspase 3 by western blotting in a first step. Protein expression of the 32-kDa procaspase-3 (Cell signaling Technology, Danvers, USA) and β-actin (ABCAM, Cambridge, USA), which served as a loading control, was determined in homogenized samples of frozen ischemic myocardial tissue.

To confirm results of western blotting we used immune fluorescence in a second step to depict active caspase 3 in the ischemic myocardial tissue. Paraffin sections were dewaxed in xylene and hydrated. Sections were heated in 10 mM sodium citrate (pH 6.0) for 1 minute at 740 W followed by 9 minutes at 370 W and for 15 minutes at 100 W. The sections were incubated overnight at 4°C with a primary rabbit antibody against cleaved caspase-3 (R&D Systems, Minneapolis, MN, USA; AF 835) which recognizes the p20/p17 subunit in the cytoplasm of apoptotic cells. The secondary goat anti-rabbit antibody (Alexa Fluor 488 SFX Kit, A 31628; Invitrogen, Paisley, UK) was labelled with green-fluorescent dye. Sections were counterstained with bisbenzimide (Sigma Aldrich, St. Louis, USA; B2883). Immunofluorescence sections were observed with a fluorescent microscope (Axiovert 200; Carl Zeiss, Jena, Germany) and photographed.

**Myeloperoxidase assay**

Myeloperoxidase, an enzyme present in leukocytes, is an index of tissue immune cell infiltration and was determined as previously described [7]. The quantification of myeloperoxidase activity in ischemic (AAR) and non-ischemic (AANR) myocardial tissue samples was performed with a myeloperoxidase assay kit (Cytostore, Calgary, Alberta, Canada) and absorbance at 450 nm was measured immediately after addition of chromogen, and again at 60 second intervals.

**Western blotting**

Two different protocols employing RIPA buffer and SDS, respectively, were used for the preparation of tissue samples. (i) protein extraction using RIPA buffer: Samples of frozen myocardial tissue (30 mg) were mixed with 800 µl RIPA buffer including protease and phosphatase inhibitors (Roche, Mannheim, Germany) and homogenized on ice with 10-15 strokes using a teflon-glass homogenizer. The solution was centrifuged for 2 minutes at 4°C and 300rpm using QIAshredder filters (Qiagen) and was stored at -20°C until use. (ii) protein extraction using SDS: Tissue samples were homogenized as described above in a buffer containing 2% sodium dodecyl sulfate (SDS), 10 mM Tris-HCl (pH 7.4) freshly supplemented with sodium fluoride (10 mM), sodium pyrophosphate (10 mM), sodium orthovanadate (1 mM), sodium molybdate (1 mM), phenylarsine oxide (1 M), and aprotinin (10 μg/ml). The homogenate obtained by both methods was boiled for 5 minutes after addition of SDS-polyacrylamide gel electrophoresis (PAGE) sample buffer. Protein concentration was determined in a homogenate aliquot with a BCA Protein Assay kit (Pierce Biotechnology, Rockford, USA). An equal amount of protein (30 μg) of each sample was separated by 12% SDS-PAGE and transferred onto a nitrocellulose membrane (Amersham Pharmacia Biotech, Piscataway, USA). The membranes were then incubated in a blocking solution of phosphate buffered saline (PBS), pH 7.4, containing 5% non-fat milk powder for 1 hour at room temperature followed by overnight incubation with specific antibodies for procaspase-3 (Cell signaling Technology, Danvers, USA; dilution 1:200) and β-actin (ABCAM, Cambridge, USA), which served as a loading control. After washing in PBS containing 0.05% Tween 20 (PBS-T; Sigma-Aldrich, St. Louis, USA), the membranes were incubated for 1 hour with peroxidase-conjugated goat antirabbit immunoglobulin G (Amersham Pharmacia Biotech, Piscataway, USA). The final reaction was visualized using enhanced chemiluminescence (ECL Western Blotting Detection Reagents, Amersham Pharmacia Biotech, Piscataway, USA), and the membranes were exposed to x-ray film. Bands were scanned and the optical density was quantified with densitometry and expressed as the ratio of procaspase 3 to β-actin.

**Quantitative real-time RT-PCR**

Transcript levels of interleukin (IL)-1β, IL-6, IL-10, tumor necrosis factor (TNF)-α and intercellular adhesion molecule (ICAM)-1 were investigated in ischemic and non-ischemic cardiac tissue of all surviving animals and compared with tissue of control animals. Gene expression levels were determined using quantitative real-time reverse transcriptase polymerase chain reaction (RT-PCR). Total RNA was isolated from frozen tissue using a commercially available kit (RNeasy Mini Kit; Qiagen, Hilden, Germany). RNA yield and purity were measured by a spectrophotometer (Spectronic GENESYS 10UV, Rochester, USA). 500 ng RNA was subsequently used for the synthesis of first-strand cDNA with random hexamers using the MultiScribe™ Reverse Transcriptase System (Applied Biosystems, Foster City, USA) according to the manufacturer’s instructions. Integrity of RNA was determined by gel electrophoreses on a standard 2% agarose gel stained with ethidium bromide and visualized by exposure to ultraviolet light. Quantitative PCR was performed utilizing the TaqMan Universal PCR Master Mix (Applied Biosystems, Foster City, USA). Genes of interest were amplified using custom primers and probes. All reactions were performed on the ABI Prism 7900HT Sequence Detection System (Applied Biosystems, Foster City, USA) utilizing the following conditions: Stage 1, 2 minutes at 50°C; stage 2, 10 minutes at 95°C; stage 3, 45 cycles of 15 seconds of melting at 95°C followed by DNA synthesis for 1 minutes at 60°C. Stable standard gene expression for internal standardisation of target gene expression data was determined by BestKeeper application [8]. Out of three candidates, β-actin, HPRT, and glyceraldehyde-3-phosphate dehydrogenase (GAPDH), GAPDH demonstrated least variations. Thus, all samples were normalized for input based on GAPDH.

Intron spanning primers and minor groove binder probes used for quantitative RT-PCR were purchased as Assay-on-Demand from Applied Biosystems (Nieuwekerk a/d IJsel, The Netherlands). Primers were designed using primer design software [9]. Primers were analyzed using Oligo Analysis & Plotting Tools software from Operon, in order to avoid secondary structures such as hairpins and loops. Primer sequences and amplicon lengths are shown in Table S1. Serial cDNA dilution curves were produced to calculate the amplification efficiency for all genes. A graph of threshold cycle (Ct) versus log10 relative copy number of the sample from a dilution series was produced. The slope of the curve was used to determine the amplification efficiency: efficiency = 10 (-1/slope) [10].

Data analysis was performed according to a relative standard curve method using an Excel spreadsheet, and statistical significance was tested using randomization testing, as provided in the REST2005 program [11]. Samples with a probability value of < 0.05 were regarded to be significant different between groups. The ratio of the target gene expression level was determined for each sample, and the results are expressed as x-fold increase over sham.

***In-vitro* cell culture**

HT-1080 cells [12] (European Collection of Cell Cultures, Salisbury, U.K.) were grown in DMEM/HAM’S F-12 medium (PAA, Coelbe, Germany) supplemented with 0.25% (v/v) NaHCO_3_, 4 mM L-glutamine, 100 U/ml penicillin, 100 µg/ml streptomycin (all from Seromed, Berlin, Germany) and 10% FCS (Linaris, Bettingen, Germany). Cells from passages between 6 and 9 were used for the experiments. Cells were seeded at 3 x 10^5^ cells in T-75 flasks, at 5 x 10^4^ cells/well in 6-well plates or at 2 x 10^3^ cells/well in 96-well plates, respectively (purchased from Falcon, Heidelberg, Germany). Cultures were maintained in a humidified incubator at 37°C in 5% CO_2_. Subculturing was done by trypsinization (0.025% trypsin - 1 mM EDTA; Bioproducts, Ingelheim, Germany) for 2-3 minutes at 37°C. Cell counts were performed with a hemocytometer. For hormone-challenge experiments one dose of human recombinant IL-1β (Invitrogen, Carlsbad, USA) was added to the FCS containing culture media and stimulations were performed between 3 hours (RT-PCR) and 48 – 96 hours (zymography, cell proliferation) without changing the medium.

**Cell proliferation assays**

MTS assays were purchased from Promega (Mannheim, Germany) and performed according to the manufacturer’s protocol. Cells were seeded at confluence of 30 to 40 % in 96 well plates with DMEM/HAM’S F-12 medium containing 10% FCS. After 24 hours, stimulation was performed with/without 1, 10 and 100 ng/ml IL-1β in medium containing 10% FCS. Assays were performed 72 hours after stimulation. Each experiment was repeated two times using 8 samples per group.

**Isolation of RNA, RT-PCR and semiquantitative PCR**

Cells were grown to subconfluence in DMEM/HAM’S F-12 medium containing 10% FCS for 24 hours. The following day cells were treated with/without human recombinant IL-1β (10 ng/ml). Cells were washed twice with PBS (Sigma Aldrich, Hamburg, Germany) and suspended in RLT buffer (Qiagen GmbH, Hilden, Germany) containing 1% beta-mercaptoethanol (according to manufacturer’s protocol). Isolation of RNA was done with Qiagen RNeasy minikit, followed by reverse transcription using random hexamer primers. 200 ng of total RNA was used for further semiquantitative RT-PCR experiments. Primer sequences and amplicon sizes are displayed in Table S2. PCR products were visualized by ethidiumbromide staining in agarose gels. Negative controls were performed by omitting the respective input cDNA.

**Gelatine zymography**

Zymography was performed as described previously [13]. Briefly, cells were stimulated with IL-1β as described above and culture supernatants were collected after 48 and 96 hours. Following a 1 minute centrifugation step at 1000g, supernatants were stored at –20°C until use. Prior to gelatine zymography, samples were concentrated 5-fold using 10kDa Microcon centrifugal filter devices YM-10 (Millipore, Billerica, USA) and 30 µg protein (evaluated by BioRad protein DC assay) was loaded and separated on 7 % SDS – page gels (containing 1 mg/ml gelatine) under non-reducing conditions.

For detection of matrix metalloproteinase (MMP) activity in-vivo, myocardial tissue samples were homogenized in RIPA buffer (for details see Western blotting) and treated like the cell culture supernatants with the exception that no concentration step was performed. After electrophoresis the gels were soaked in 2.5 % Triton X-100 for 30 minutes to remove SDS and incubated in Tris-HCl (50 mmol/l, pH 7.5), containing CaCl_2_ (5 mmol/l), and ZnCl_2_ (1 mmol/l) overnight at 37°C. After Coomassie blue staining white bands of lysis indicated digestion of gelatine by MMPs. To account for the intrinsic MMP activity within the culture medium and to compare the MMP activities on different days with or without IL-1β stimulation, densitometric analysis was performed using the ImageJ 1.41 software (ImageJ, NIH, USA).

**REFERENCES**

1. Roesner JP, Petzelbauer P, Koch A, Mersmann J, Zacharowski PA, et al. (2007) The fibrin-derived peptide Bbeta15-42 is cardioprotective in a pig model of myocardial ischemia-reperfusion injury. Crit Care Med 35: 1730-1735.

2. (2005) 2005 American Heart Association Guidelines for Cardiopulmonary Resuscitation and Emergency Cardiovascular Care. Circulation 112: IV1-203.

3. Idris AH, Becker LB, Ornato JP, Hedges JR, Bircher NG, et al. (1996) Utstein-style guidelines for uniform reporting of laboratory CPR research. A statement for healthcare professionals from a task force of the American Heart Association, the American College of Emergency Physicians, the American College of Cardiology, the European Resuscitation Council, the Heart and Stroke Foundation of Canada, the Institute of Critical Care Medicine, the Safar Center for Resuscitation Research, and the Society for Academic Emergency Medicine. Writing Group. Circulation 94: 2324-2336.

4. Tei C, Ling LH, Hodge DO, Bailey KR, Oh JK, et al. (1995) New index of combined systolic and diastolic myocardial performance: a simple and reproducible measure of cardiac function--a study in normals and dilated cardiomyopathy. J Cardiol 26: 357-366.

5. Gottdiener JS, Bednarz J, Devereux R, Gardin J, Klein A, et al. (2004) American Society of Echocardiography recommendations for use of echocardiography in clinical trials. J Am Soc Echocardiogr 17: 1086-1119.

6. Abbate A, Biondi-Zoccai GG, Baldi A (2002) Pathophysiologic role of myocardial apoptosis in post-infarction left ventricular remodeling. J Cell Physiol 193: 145-153.

7. von Bismarck P, Klemm K, Wistadt CF, Winoto-Morbach S, Uhlig U, et al. (2007) Surfactant "fortification" by topical inhibition of nuclear factor-kappaB activity in a newborn piglet lavage model. Crit Care Med 35: 2309-2318.

8. Pfaffl MW, Tichopad A, Prgomet C, Neuvians TP (2004) Determination of stable housekeeping genes, differentially regulated target genes and sample integrity: BestKeeper--Excel-based tool using pair-wise correlations. Biotechnol Lett 26: 509-515.

9. Rozen S, Skaletsky H (2000) Primer3 on the WWW for general users and for biologist programmers. Methods Mol Biol 132: 365-386.

10. Pfaffl MW (2001) A new mathematical model for relative quantification in real-time RT-PCR. Nucleic Acids Res 29: e45.

11. Pfaffl MW, Horgan GW, Dempfle L (2002) Relative expression software tool (REST) for group-wise comparison and statistical analysis of relative expression results in real-time PCR. Nucleic Acids Res 30: e36.

12. Rasheed S, Nelson-Rees WA, Toth EM, Arnstein P, Gardner MB (1974) Characterization of a newly derived human sarcoma cell line (HT-1080). Cancer 33: 1027-1033.

13. Kleiner DE, Stetler-Stevenson WG (1994) Quantitative zymography: detection of picogram quantities of gelatinases. Anal Biochem 218: 325-329.
