# Supplementary material for: Hypothermia and Postconditioning after Cardiopulmonary Resuscitation Reduce Cardiac Dysfunction by Modulating Inflammation, Apoptosis and Remodeling
Source: PLoS One. 2009 Oct 26;4(10):e7588. doi: 10.1371/journal.pone.0007588 (PMC2764338; doi:10.1371/journal.pone.0007588)
Supplement: Table S1 — Primer sequences and TaqMan probes (in-vivo experiment). (0.06 MB DOC) [file pone.0007588.s002.doc]

**Table S1. Primer sequences and TaqMan probes (in-vivo experiment).**

| Target | Forward | Backward | TaqMan | Reporter | Quencher |
| --- | --- | --- | --- | --- | --- |
| GAPDH | attgccctcaacgaccact | ggcctctctcctcctcgc | tccaccaccctgttgctgtagccaaat | FAM | TAMRA |
| HPRT | cggcctccgttatggcg | ggtcataacctggttcgtcatca | cgcagccccagcgtcgtgatta | FAM | TAMRA |
| βAktin | tcatcaccatcggcaacg | ttcctgatgtccacgtcgc | ccttcctgggcatggagtcctgc | FAM | TAMRA |
| IL-1β | atgctgaaggctctccacct | ttgttgctatcatctccttgcac | aagctcatgcagaacaccacttctctcttcaagt | FAM | TAMRA |
| IL-6 | gaactccctctccacaagcg | gggtagggaaggcagtagcc | cttcagtccagtcgccttctccctgg | FAM | TAMRA |
| IL-10 | gctggaggactttaagggttacc | atatcctcccatcactctctgc | ttgccaagccttgtcagagatgatccag | FAM | TAMRA |
| TNF-α | ggcccaaggactcagatca | cggctttgacattggctaca | caaacctcagataagcccgtcgcc | FAM | BBQ |
| ICAM-1 | ctggcagacgagaaggtggt | gctcgctcagggtcaggtt | tgaccttctacagcttcccacctccca | FAM | TAMRA |

Primer sequences and TaqMan probes of the cytokines interleukin (IL)-1β, IL-6, IL-10, tumor necrosis factor (TNF)-α and intercellular adhesion molecule (ICAM)-1. GAPDH indicates glyceraldehyde-3-phosphate dehydrogenase ; HPRT, hypoxanthin-guanin-phosphoribosyltransferase.
