# Supplementary material for: Hypothermia and Postconditioning after Cardiopulmonary Resuscitation Reduce Cardiac Dysfunction by Modulating Inflammation, Apoptosis and Remodeling
Source: PLoS One. 2009 Oct 26;4(10):e7588. doi: 10.1371/journal.pone.0007588 (PMC2764338; doi:10.1371/journal.pone.0007588)
Supplement: Table S2 — Primer sequences and amplicon sizes (in-vitro cell culture). (0.04 MB DOC) [file pone.0007588.s003.doc]

**Table S2**. Primer sequences and amplicon sizes (in-vitro cell culture).

| Target | Forward | Backward | Amplicon size, bp |
| --- | --- | --- | --- |
| IL-1β | gaggatgacttgttctttgaag | gttgctcatcagaatgtgggag | 1096 |
| IL-1β receptor | atgagacaatggaagtagac | tagatgaaaacagaacacac | 372 |
| MMP-2 | ggcacccatttacacctacaccaa | gcttccaaacttcacgctcttcag | 694 |
| MMP-9 | accgctatggttacactcgg | gcaggcagagtaggagcg | 584 |
| 18s rRNA | gttggtggagcgatttgtctgg | agggcagggacttaatcaacgc | 348 |

Primer sequences of interleukin (IL)-1β, IL-1β receptor, matrix metalloproteinase (MMP)-2 and -9, and 18s rRNA.
