# Supplementary material for: Hypothermia and Postconditioning after Cardiopulmonary Resuscitation Reduce Cardiac Dysfunction by Modulating Inflammation, Apoptosis and Remodeling
Source: PLoS One. 2009 Oct 26;4(10):e7588. doi: 10.1371/journal.pone.0007588 (PMC2764338; doi:10.1371/journal.pone.0007588)
Supplement: Results S1 — Preliminary results as Online Supplement and Figure legends for Supporting Information file - Figure S1. (0.05 MB DOC) [file pone.0007588.s004.doc]

# **Supporting information file -**

**Results S1**

# Hypothermia and postconditioning after cardiopulmonary resuscitation reduce cardiac dysfunction by modulating inflammation, apoptosis and remodeling

***In-vitro* cellular effects of IL-1β on proliferation and MMPs activity**

IL-1β was one of the cytokines that was strongly upregulated on gene- and protein levels following cardiopulmonary resuscitation and myocardial ischemia, and attenuated by HT and HT+SEV in our study (Figure 5A-C). As MMPs are involved in myocardial remodeling, and MMP-9 activity was also attenuated by HT and HT+SEV, we decided to elucidate the regulatory effects of IL-1β on MMPs activity and cell proliferation - two major events in tissue remodeling - employing the well characterized human fibroblast cell line HT-1080. Using RT-PCR experiments we found that HT-1080 cells express IL-1β and the IL-1 receptor type I, and that stimulation with IL-1β (10 ng/ml for 3 hours) leads to a moderate increase in mRNA-levels of IL-1β and the IL-1 receptor (Figure S1A). Stimulation with different concentrations of IL-1β resulted in a significant increase in cell numbers after 72 hours, with 10 ng/ml IL-1β having the most distinct effect (Figure S1B). Interestingly, expression of MMP-2 and MMP-9 mRNA was strongly induced by 10 ng/ml IL-1β, whereas MMP-2 and MMP-9 mRNAs were not detectable or only found at low expression levels using RT-PCR experiments in unstimulated HT-1080 cells. Amplification of 18S-rRNA was performed to evaluate differences in applied cDNA amounts (Figure S1C). Gelatine zymography performed with supernatants of HT-1080 cells grown 48 and 96 hours in medium containing 10% FCS revealed activity of MMP-2 and MMP-9 in all samples, including the FCS containing culture medium. Stimulation with 10 ng/ml IL-1β, however, resulted in an increased activity of both MMPs in the treated cultures (Figure S1D).

**Figure S1. Effects of IL-1β on cell proliferation and MMPs activity in vitro.** Human HT-1080 cells were grown as described in Section I. *A)* Unstimulated and IL-1β treated cells express IL-1β and the IL-1 receptor type I as shown by RT-PCR. Amplification of 18s rRNA was performed to evaluate differences in applied cDNA amounts, negative controls were performed by omitting the respective cDNA. *B)* Subconfluent cultures were stimulated with IL-1β and subjected to colorimetric cell proliferation assays. Application of IL-1β to the cultures resulted in a dose-dependent statistically significant increase in cell numbers after 72 hours. Experiments were independently performed two times, with 8 samples per experiment, one representative experiment is shown. *p<0.05, #p<0.001 vs. control. *C)* Matrix metalloproteinase (MMP)-2 and MMP-9 mRNAs were not detectable or only found at low expression levels using RT-PCR experiments in unstimulated HT-1080 cells. IL-1β at a concentration of 10 ng/ml for 3 hours induced gene expression of both MMPs. Amplification of 18S-rRNA was performed to evaluate differences in applied cDNA amounts. *D)* Gel zymography performed with supernatants of HT-1080 cells grown 48 and 96 hours in medium containing 10 % FCS revealed activity of MMP-2 and MMP-9 in all samples, including the FCS containing culture medium. Stimulation with 10 ng/ml IL-1β for 96 hours, however, resulted in an increased activity of both MMPs in the treated cultures.
