# Supplementary figures and images for: Hypothermia and Postconditioning after Cardiopulmonary Resuscitation Reduce Cardiac Dysfunction by Modulating Inflammation, Apoptosis and Remodeling
Source: PLoS One. 2009 Oct 26;4(10):e7588. doi: 10.1371/journal.pone.0007588 (PMC2764338; doi:10.1371/journal.pone.0007588)

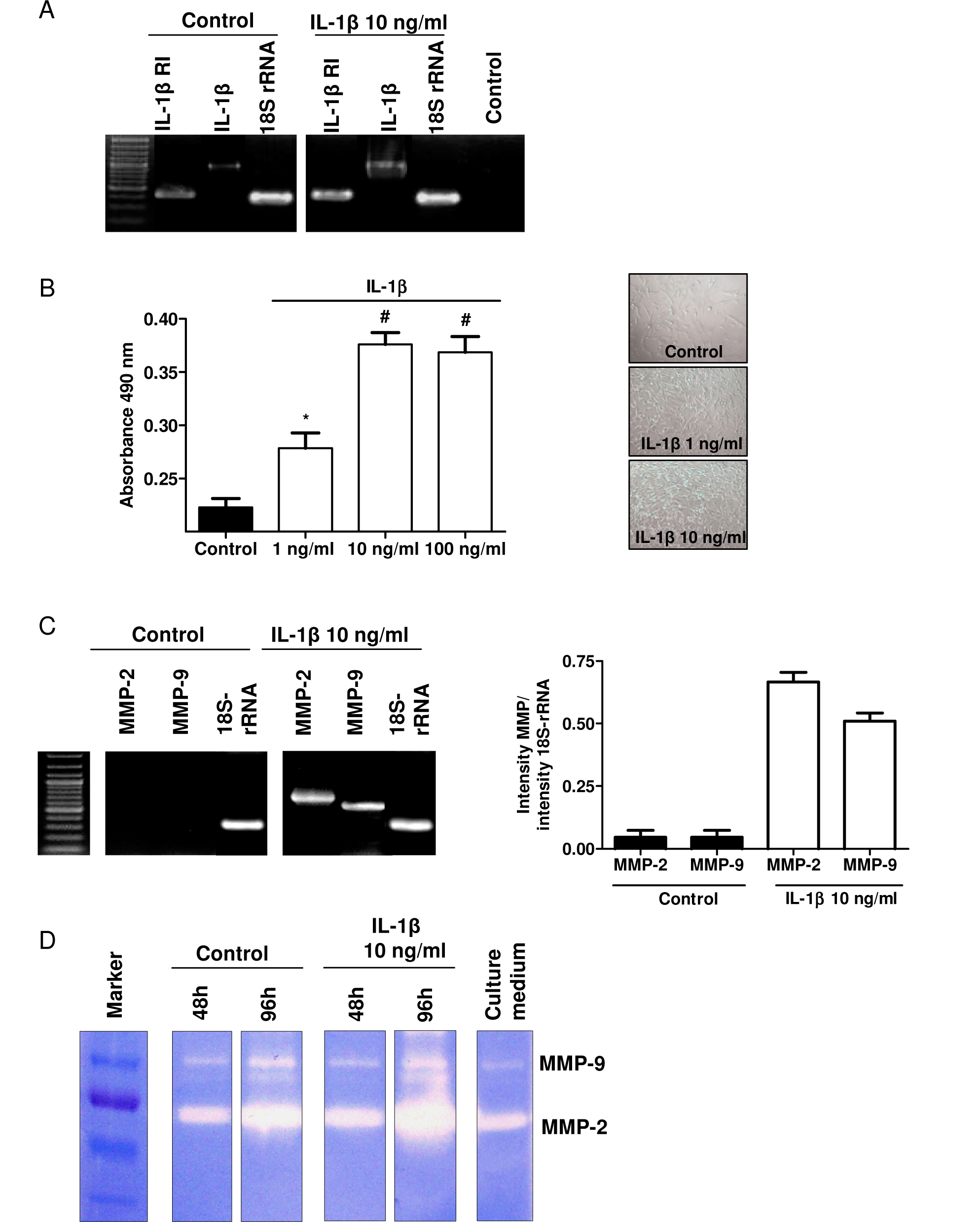

Supplement: Figure S1 — Effects of IL-1β on cell proliferation and MMPs activity in vitro. (2.37 MB TIF) [file pone.0007588.s005.tif]
